# Local Treatment of Hydrogen-Rich Saline Promotes Wound Healing In Vivo by Inhibiting Oxidative Stress via Nrf-2/HO-1 Pathway

**DOI:** 10.1155/2022/2949824

**Published:** 2022-03-08

**Authors:** Yujie Li, Chengcheng Shen, Xin Zhou, Jianghe Zhang, Xiaoyue Lai, Yiming Zhang

**Affiliations:** ^1^Department of Plastic and Cosmetic Surgery, Xinqiao Hospital, Army Medical University, Chongqing 400037, China; ^2^Department of Dermatology, The First Affiliated Hospital of Chongqing Medical University, Chongqing 400016, China; ^3^Department of Cell Biology, Army Medical University, Chongqing 400037, China; ^4^Department of Ultrasound, Xinqiao Hospital, Army Medical University, Chongqing 400037, China

## Abstract

Wound healing is a complex dynamic process involving a large number of biological events. Excessive oxidative stress is a key factor delaying wound healing. Hydrogen is an antioxidant, anti-inflammatory, and antiapoptotic medical gas with safety, effectiveness, and penetrability. However, the effects of local treatment of hydrogen on wound healing and its potential mechanisms remain unclear. In this study, Kunming (KM) mice were used to set up a wound model. All the mice were randomly divided into the control, the local treatment with saline group, the local treatment with the hydrogen-rich saline group, and the intraperitoneal injection of the hydrogen-rich saline group. To evaluate the impact of hydrogen-rich saline on wound healing, we assessed the wound healing rate, wound closure time, histomorphology, oxidative stress indicators, inflammatory cytokines, the apoptosis index, and the expression of the nuclear factor-erythroid-related factor 2(Nrf-2). Furthermore, the immortalized nontumorigenic human epidermal (HaCaT) cells were chosen to investigate the therapeutic effects of hydrogen-rich medium on oxidative stress and its underlying mechanisms. The results showed that local treatment of hydrogen-rich saline shortened wound closure time and reduced the level of proinflammatory cytokines and lipid peroxidation. Meanwhile, it decreased the cell apoptosis index and increased the Nrf-2 expression. Besides, hydrogen-rich medium relieved the oxidative stress via the activation of the Nrf-2/heme oxygenase-1 (HO-1) pathway. In conclusion, local treatment of hydrogen-rich saline exhibits the healing-promoting function through antioxidant, anti-inflammatory, and antiapoptotic effects. Hydrogen relieves the oxidative stress in the wound microenvironment via Nrf-2/HO-1 signaling pathway. This study may offer a new strategy to promote wound healing and a new perspective to illustrate the mechanism of wound healing.

## 1. Introduction

With the development of society and the change of human disease spectrum, acute and chronic wounds caused by diseases and injuries are increasing. According to published statistics, the prevalence of chronic wounds in the general population is 2.21‰ [1]. In addition, promoting the wound healing process after aseptic operation (e.g., cosmetic surgery) is of great significance to reduce the incidence of related complications. Therefore, it is vital to find an effective and convenient method to promote the healing of acute and chronic wounds in clinic.

Wound healing is a dynamic, complex, and ordered biological process, including the joint participation of a variety of cells, growth factors, mediators, extracellular matrix components, and the structures of the extracellular matrix. All these factors show a high degree of order, integrity, and interactivity under exquisite control [2]. It is generally believed that the wound healing process consists of hemostasis, inflammation, proliferation, and remodeling. These stages occur sequentially and overlap with no distinct boundaries [3]. In the initial stage of trauma, ischemia and hypoxia will trigger massive production of free radicals and reduction of antioxidant capacity [4]. These events can easily lead to lipid peroxidation, protein oxidation, DNA damage, and even severe cell and tissue damage, which would delay the wound healing eventually [5, 6]. Moreover, a series of exuberant cellular activities (like cell migration, movement, and secretion) occur in the wound healing process, which requires a significant increase in cell oxygen consumption to provide extra energy. Subsequently, more reactive oxygen species (ROS) are produced [6, 7]. While in the late stage of wound healing, the ischemia and hypoxia conditions are improved with the closure and vascularization of the wound. In this stage, oxidative stress responses tend to be mild, as remodeling and maturation become the dominant processes.

Oxidative stress can activate macrophages and neutrophils to release inflammatory mediators such as interleukins (IL) and tumor necrosis factors (TNF) [8]. On the one hand, they can interact with inflammatory cells, resulting in telangiectasia, hyperemia, and increased permeability and tissue edema, aggravating local inflammatory response; on the other hand, they can also activate the cascade of cytokines, hindering the process of wound healing [9]. Moreover, oxidative stress can mediate cell apoptosis through the mitochondrial pathway, the endoplasmic reticulum stress pathway, and the death receptor pathway [10]. Therefore, proper regulation of oxidative stress in wound microenvironment may play a positive role in promoting wound healing. Recent researches reported that Nrf-2 plays a vital role in alleviating oxidative stress and promoting wound healing [11, 12]. The Nrf-2 signal pathway participates in the regulation of antioxidative gene expression and thus improves the cell protection [13].

Hydrogen is an anti-inflammatory, antioxidant, and antiapoptotic medical gas with safety and effectiveness. By neutralizing hydroxyl radical and peroxynitrite ion, hydrogen can protect cells and tissues from oxidative stress and inflammatory damage [14]. Media which contain hydrogen, such as hydrogen-rich saline and hydrogen-rich medium, can work in similar way as hydrogen itself. Previous studies have shown that inhaling hydrogen, drinking hydrogen-rich water, and intraperitoneal injection of hydrogen-rich saline can be efficacious in treating ischemia-reperfusion injury, sepsis, diabetes, radiation injury, and other diseases [15–19]. Latest researches suggest that hydrogen can also be beneficial on skin injury. For example, the apoptotic cell index of radiation injured rats was significantly reduced through inhalation of hydrogen [20], the damage of psoriasis was alleviated after hydrogen-rich water bath [21], and the oral wound healing in rats was accelerated after drinking hydrogen-rich saline [22].

Based on the above, this study aims at investigating the effect of local treatment of hydrogen-rich saline on wound healing through the establishment of mouse wound model and seeks its potential molecular mechanisms via cell experiments. This study is also trying to offer a new strategy to promote wound healing and a new perspective to illustrate the mechanisms of wound healing.

## 2. Materials and Methods

### 2.1. Preparation of Hydrogen-Rich Saline and Hydrogen-Rich Medium

High-purity hydrogen was produced by a multifunctional hydrogen machine (FH-600, Haowei Experimental instrument Co., Ltd, China). Hydrogen-rich saline was prepared using the method reported by Professor Sun Xuejun, Department of Marine Medicine, Naval Medical University [23–25]. Extract 50 mL solution from 100 mL bagged saline to get a half-empty saline bag. Inject hydrogen into the half-empty saline bags under 0.4 MPa and keep the gassed bags in 4°C. After 24 hours, replace the gas with fresh hydrogen and then sterilize with ultraviolet light. The methylene blue solution containing colloidal platinum (MIZ, Japan) was used to confirm that the hydrogen concentration in the hydrogen-rich saline was 0.7-0.8 mmol/L [26]. To make hydrogen-rich medium, 10% fetal bovine serum (Hyclone, US), 89% MEM (Hyclone, US), and 1% penicillin/streptomycin were infused into sterile empty 100 mL saline bag [27, 28], and then pump the hydrogen into the bag under 0.4 MPa and keep it in 4°C. The remaining steps are the same as the preparation of hydrogen-rich saline. In order to guarantee the concentration of hydrogen, all reagents were used right after preparation and the saline bags filled with the hydrogen-rich medium were all sealed in aluminum bags during preservation.

### 2.2. Establishment of the Full-Thickness Skin Wound Model and Grouping

All animal experiments were reviewed and approved by the Animal Ethics Committee of the Army Medical University, and all procedures performed on the animals were conducted according to the guidelines of ARRIVE and the US NIH [29]. 8-week-aged male KM mice weighing from 29 g to 35 g were purchased from the Experimental Animal Center of Xinqiao Hospital, Army Medical University. 48 mice were randomly divided into the control, the local treatment of the saline group (LTS group), the local treatment of the hydrogen-rich saline group (LTH group), and the intraperitoneal injection of the hydrogen-rich saline group (IIH group). No treatment was taken in the control. For the LTS group, sterile saline was used three times a day to smear the wounds on both dorsal sides. For the LTH group, the wounds were smeared with hydrogen-rich saline three times a day. For the IIH group, the mice were intraperitoneally injected with 0.3 mL hydrogen-rich saline once a day.

The full-thickness skin wound model was created as described. Mice were anesthetized by intraperitoneal injection with 1% pentobarbital sodium. Dorsal hairs of the mice were shaved by electric razor and carefully removed from the roots of the hair follicles with hair remover cream. They were then put back to the cage for rest after total clearance by warm water. The next day, after anesthetization, sterile perforator with a diameter of 8 mm was used to create wounds on the dorsal skin, so that each mouse had two full-thickness skin wounds to the depth of fascia layer. All mice were housed individually after awakening from anesthetization and then raised under the pathogen-free condition with free access to food and water. The ambient temperature was maintained within 23-27°C.

### 2.3. Wound Healing Assessment and HE Staining

The wound was traced and recorded by digital camera once a day. The wound closure time was defined as the time when epithelialization was completed. Afterwards, the wound area was measured by the ImageJ software. The wound healing rate = (the original wound area—the unhealed wound area)/the original wound area.

On the 3rd, 7th, and 12th days after the establishment of the wound model, same number of mice in each group were euthanized after anesthesia. The wound tissue was fixed by paraformaldehyde and embedded in paraffin and then sliced in 4 *μ*m. After dewaxed and rehydrated, the slices were stained by HE staining kit (C0105M, Beyotime, China). These slices were observed and photographed under 40x and 100x magnifications (BX63, Olympus, JAPAN).

### 2.4. Assessment of Oxidative Stress

On the 7th day after the establishment of the wound model, three mice in each group were euthanized after anesthesia. In order to evaluate the level of lipid peroxidation, malondialdehyde (MDA) assay detection kit (BC0025, Solarbio, Beijing, China) was used to detect the content of MDA in wound tissue. The results were expressed in nmol/g. Meanwhile, superoxide dismutase (SOD) activity detection kit (BC0175, Solarbio, Beijing, China), reduced glutathione (GSH) assay detection kit (BC1175, Solarbio, Beijing, China), and catalase (CAT) activity detection kit (BC0205, Solarbio, Beijing, China) were used to evaluate the oxidative stress level of wound tissues. The results of SOD and CAT were expressed in U/g while GSH was expressed in *μ*g/g. The absorbance was measured by the Thermo Scientific Varioskan Flash full wavelength scanning multifunctional reader (Thermo, US).

### 2.5. Assessment of Inflammatory Cytokines

Quantitative real-time PCR (qPCR) was used to detect the expression of inflammatory factor-related genes in wound tissue. The total RNA extraction kit (LS1040, Promega, Shanghai, China), the reverse transcription kit (A5001, Promega, Shanghai, China), and the qPCR kit (A6001, Promega, Shanghai, China) were used perform total RNA extraction, reverse transcription, and qPCR, respectively, following the instructions of the manufacturers. The reverse transcription process was accomplished by a PCR apparatus (Mycycler, Bio-rad, US), and the qPCR process was accomplished by ViiD7 real-time PCR System (Applied Biosystems, US). The primers were synthesized by Shenggong Bioengineering Co., Ltd (Shanghai, China). The sequence of each gene primer is shown in Table 1.

### 2.6. TUNEL Staining

The paraffin slices of the wound tissue were stained following the instruction of the In Site Cell Death Detection Kit (Roche, Switzerland). The stained slices were observed and photographed by a fluorescence microscope (IX83, Olympus, JAPAN). Three slices were randomly selected from each group and three visual fields were analyzed in each slice. The number of apoptotic cells was counted by the ImageJ software. The apoptosis index (AI) = the number of apoptotic cells/the number of total cells.

### 2.7. Immunohistochemistry Assay

Immunohistochemical staining was performed in the paraffin-embedded tissue slices for the detection of nuclear factor-erythroid-related factor 2 (Nrf-2). After dewaxed with xylene and washed in phosphate buffer saline (PBS) for 10 min, the slices were immersed into 0.01 mol/L citrate buffer (pH = 6) at 95°C for 15 min to repair antigens. Afterwards, the slices were incubated with 3% hydrogen peroxide for 15 minutes and then blocked with goat serum at 23°C for 15 min. Anti-Nrf-2 antibody (1 : 100, AB137550, Abcam, Cambridge, England) was added and incubated with slices at 4°C overnight. After that, the slices were incubated with goat antirabbit secondary antibody (PV6001, ZSGB-BIO, Beijing, China) and then developed by diaminobenzidine (DAB) kit (AR1022, Boster, Wuhan, China). Finally, the slices were observed and photographed by a microscope (BX63, Olympus, JAPAN) at 400x magnification. The positive staining was counted and analyzed by the ImageJ software.

### 2.8. Cell Culture and Grouping

HaCaT cells (DingGuo ChangSheng Biological Co., Ltd, Beijing, China) were cultured in standard medium following the instructions. The standard medium contains 10% fetal bovine serum (Hyclone, US), 89% MEM medium (Hyclone, US), and 1% penicillin/streptomycin. The cells were incubated at 37°C in a humidified atmosphere of 5% CO_2_ and 95% air in a cell incubator.

In ROS fluorescence detection and corresponding cell viability analysis, HaCaT cells after standard cultivation were divided into five groups. The control: cultured in standard medium without H_2_O_2_ stimulation; Standard medium+H_2_O_2_ group (SM+H_2_O_2_ group): cultured in standard medium and then given H_2_O_2_ stimulation; Hydrogen-rich medium group (HM group): cultured in hydrogen-rich medium without H_2_O_2_ stimulation; Hydrogen-rich medium+H_2_O_2_ group (HM+H_2_O_2_ group): cultured in hydrogen-rich medium and then given H_2_O_2_ stimulation; and Resveratrol+H_2_O_2_ group (Res+H_2_O_2_ group): cultured in standard medium and then given 10 *μ*mol/L resveratrol and stimulated by H_2_O_2_.

In cellular immunofluorescence detection and corresponding cell viability analysis, in order to confirm whether Nrf-2 has participated in the process of antioxidation, we used ML385 (Selleck, US), a specific inhibitor of Nrf-2 [30, 31]. HaCaT cells cultured by the standard procedure were divided into four groups. The control: incubated in standard medium for 12 hours; Standard medium+ML385 group (SM+ ML385 group): incubated with 10 *μ*mol/L ML385 in standard medium for 12 hours; Hydrogen-rich medium group (HM group): incubated with hydrogen-rich medium for 12 hours; and Hydrogen-rich medium+ML385 group (HM+ML385 group): incubated with 10 *μ*mol/L ML385 in hydrogen-rich medium for 12 hours. All groups were given H_2_O_2_ stimulation for 4 hours before following tests.

### 2.9. H_2_O_2_-Mediated Oxidative Stress and Cell Viability Analysis

Oxidative stress induced by H_2_O_2_ on HaCaT cells is a widely used model [32–34]. In order to find the appropriate concentration of H_2_O_2_ for oxidative stress stimulation on HaCaT cells, 0 *μ*mol/L, 50 *μ*mol/L, 100 *μ*mol/L, 150 *μ*mol/L, 200 *μ*mol/L, 250 *μ*mol/L, and 500 *μ*mol/L were set in our experiment. The stimulation time was 4 hours. The cell viability was detected by CCK-8 kit (C0042, Beyotime, China). Suggested by the instructions, the cells were incubated with enhanced CCK-8 solution for 1 hour, and the absorbance was measured by a microplate reader (Thermo Scientific Varioskan Flash full wavelength scanning multifunction reader, Thermo, US).

### 2.10. Cellular Immunofluorescence and ROS Fluorescence Detection

Immunofluorescence assay was performed as described [35]. In brief, the cultured adherent cells were fixed with 4% paraformaldehyde and then treated with 0.1% Trition X-100 solution to increase the cell membrane permeability. 10% goat serum was added to block at 37°C for 10 minutes, and then, the primary antibody was added individually and incubated with cells at 4°C overnight. Afterwards, the secondary antibody was added and incubated with cells at 37°C for 40 minutes. The Nrf-2 assay was performed with anti-Nrf-2 antibody (1 : 200, ab62352, Abcam, Cambridge, UK) and goat antirabbit lgG (H+L) Alexa Fluor 647 (1 : 500, A-21244, Invitrogen-IMS, US). The heme oxygenase-1 (HO-1) assay was performed with Anti-HO-1 antibody (1 : 500, abab52947, Abcam, Cambridge, UK) and goat antirabbit lgG (H + L) Alexa Fluor 488 (1 : 500, A0423, Beyotime, China). After three washes with PBS, the images were recorded by the fluorescence microscope at 400x magnification after adding an antiquenching sealing solution containing 4′,6-diamidino-2-phenylindole (DAPI, P0131, Beyotime, China). The fluorescence intensity of Nrf-2 and HO-1 was analyzed by the ImageJ software.

The 2′, 7′-dichlorofluorescein diacetate (DCFDA, ID3130, Solarbio, China) was used to detect the cellular reactive oxygen species (ROS) fluorescence. 20 *μ*mol/L DCFDA was added to the cell climbing slices. After 30 minutes of incubation, cells were observed and photographed under a fluorescence microscope (IX83, Olympus, JAPAN). In addition, 20 *μ*mol/L DCFDA was added after the cells were incubated in a 96-well plate for 12 hours. The fluorescence signal at 535 nm (excited at 482 nm) was measured immediately by the microplate reader (Thermo Scientific Varioskan Flash full wavelength scanning multifunction reader, Thermo, US). After 30 minutes of incubation, the fluorescence signal was measured again.

### 2.11. Statistical Analysis of Data

All the data are presented as mean ± SD. Comparisons between two groups were performed by two-tailed Student's *t*-test. Comparisons among multiple groups were performed by one-way ANOVA followed by the least significant difference (LSD) test. All data were statistically analyzed by the IBM SPSS Statistics 23.0 software. The charts were made by the GraphPad Prism8.0.2 software. *p* < 0.05 was considered as statistically significant.

## 3. Result

### 3.1. Hydrogen-Rich Saline Shortened the Wound Closure Time and Increased the Wound Healing Rate

**Figure 1:**
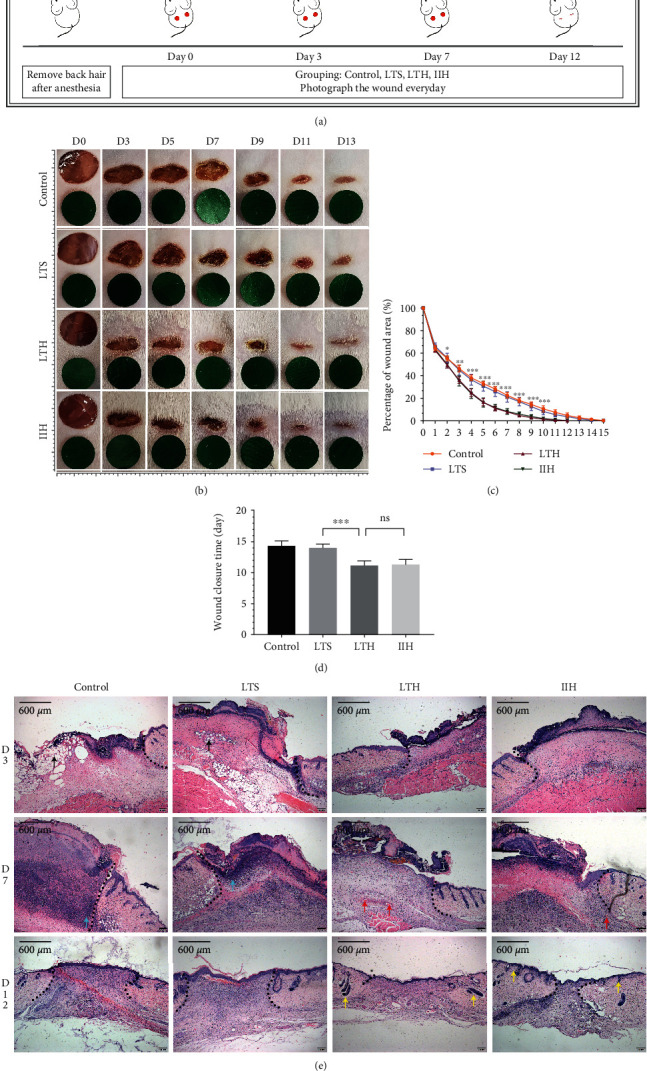
Wound healing process and HE staining. (a) Animal model establishment and time schedule for photography and sample harvest. (b) The process of wound healing. The green round sheet is a reference with a diameter of 8 mm, the same size as the D0 wound area. Each small grid in the rulers represents a length of 1 mm. (c) Daily percentage of wound area. The asterisk represents the statistically difference between the LTS group and the LTH group. (d) The wound closure time. (e) The 40x HE staining images showed different progress of wound healing in each group. The black arrows indicate edema and necrotic. The blue arrows indicate inflammatory cell infiltration. The red arrows indicate neovascularization. The yellow arrows indicate skin appendages. The black dotted line marks the edge of the wound. The results were expressed as mean ± SD, and the sample size of each group was 6. ns ^∗^*p* < 0.05, ^∗∗^*p* < 0.01, and ^∗∗∗^*p* < 0.001.

The schedule of in vivo experiment is shown in Figure 1(a). The wounds of mice were traced and recorded every day after the establishment of wound model (Figure 1(b)). Since the 3rd day, the wound areas of the control and the LTS group have been notably larger than the LTH group and the IIH group, which means the LTH group and the IIH group had higher wound healing rate (Figure 1(c)). The wound closure time of the LTH group and the IIH group was 11.16 ± 0.69 days and 11.33 ± 0.75 days, which was significantly shorter than the control (14.33 ± 0.74 days) and the LTS group (14.00 ± 0.58 days) (*p* < 0.001) (Figure 1(d)). Besides, on the 3rd day, the control and the LTS group showed more edema and necrotic tissue. Each group showed a mild degree of inflammatory cell infiltration. On the 7th day, inflammatory cell infiltration became the major manifestation instead of tissue necrosis, especially in the control and the LTS group. Neovascularization appeared in granulation tissue in the LTH and the IIH group. On the 12th day, the skin was completely epithelialized. The LTH group and the IIH group show more skin appendages like hair follicles and sebaceous than the control and the LTS group (Figure 1(e)).

### 3.2. Hydrogen-Rich Saline Treatment Mitigated the Oxidative Stress in the Wound Tissues

**Figure 2:**
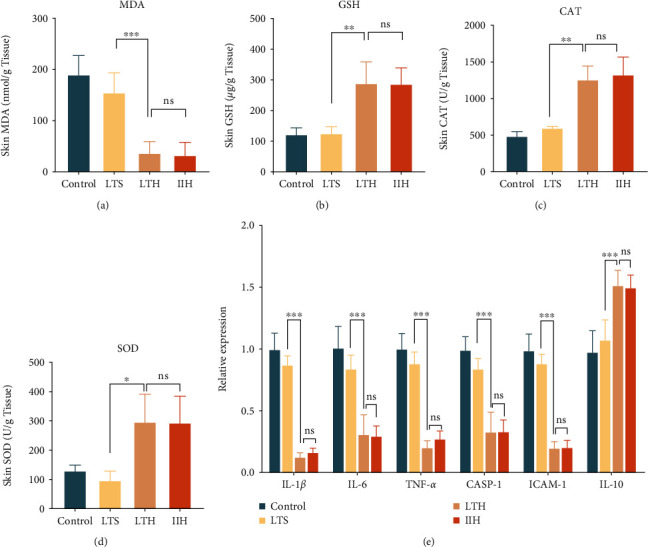
Assessment of oxidative stress and measurement of proinflammatory cytokines. (a–d) The content of MDA, GSH, CAT, and SOD in wound tissues of each group. (e) The expression of inflammatory cytokines (IL-1*β*, IL-6, TNF-*α*, CASP-1, ICAM-1, and IL-10) in wound tissues of each group. The results are expressed as mean ± SD, and the sample size of each group was 6. ns *p* > 0.05, ^∗^*p* < 0.05, ^∗∗^*p* < 0.01, and ^∗∗∗^*p* < 0.001.

Based on former literatures, SOD, MDA, CAT, and GSH were selected as predictors of oxidative stress [36–38]. The results showed that the content of MDA was higher in the control and the LTS group, indicating a higher level of lipid peroxidation. In comparison, this level was significantly reduced in the LTH group and the IIH group (Figure 2(a)). Meanwhile, in contrast to the control and the LTS group, the activity of antioxidant enzymes was profoundly improved in the LTH group and the IIH group (Figures 2(b)–2(d)). To sum up, hydrogen-rich saline treatment can mitigate the oxidative injury by reducing the level of lipid peroxidation and increasing the activity of endogenous antioxidant enzymes.

### 3.3. Hydrogen-Rich Saline Treatment Alleviated Inflammation in Wound Tissues

Through qPCR, we detected the mRNA levels of six inflammation-related indicators in the wound tissues. The results showed that the levels of IL-1*β*, IL-6, and TNF-*α* in the LTH group and the IIH group were lower than the control and the LTS group. Meanwhile, hydrogen-rich saline treatment increased the expression of IL-10, which mediates anti-inflammatory response. Cysteinyl aspartate specific proteinase-1 (Caspase-1) is a kind of protease that can activate IL-1*β* [39] and its expression in each group has similar distribution as IL-1*β* in our experiment. Intercellular cell adhesion molecule-1 (ICAM-1), a cell adhesion molecule and a member of immunoglobulins, can be expressed after induced by IL-1*β* and TNF-*α* [40]. Hydrogen-rich saline treatment can significantly reduce its expression in skin wounds. These results suggest that hydrogen-rich saline treatment can alleviate inflammatory responses by reducing the expression of proinflammatory cytokines and improving the expression of anti-inflammatory cytokines (Figure 2(e)).

### 3.4. Hydrogen-Rich Saline Treatment Reduced the Cell Apoptosis of Wound

**Figure 3:**
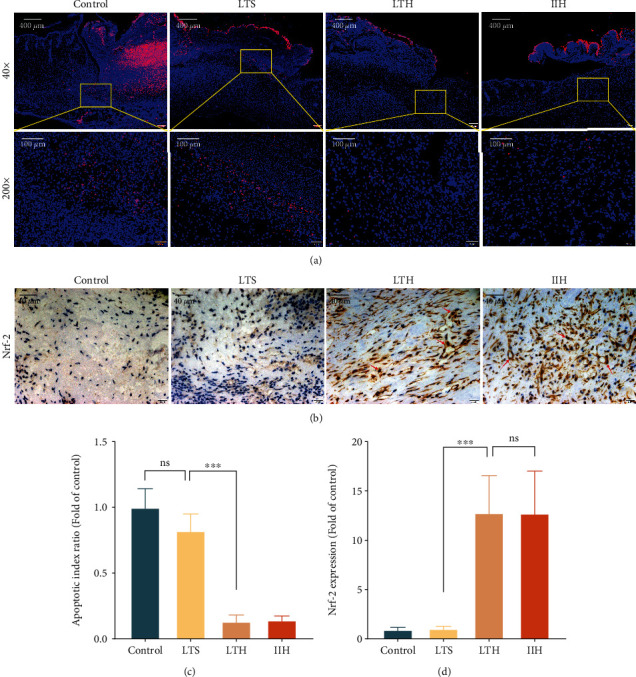
TUNEL staining for apoptosis and immunohistochemical staining for Nrf-2. (a) Each image was merged by a DAPI staining image and a TUNEL staining image. The yellow boxes in 40x images show the boundary of 200x images. Pink dots indicate apoptotic cells. (b) After immunohistochemical staining of Nrf-2, positive staining manifested as dark brown and the red arrow marked the typical positive staining overlapping with the blue nucleus (Magnification: 400x). (c) The apoptosis index ratio in each group fold of the control. (d) The ratio of positive staining in each group fold of the control. The results are expressed as mean ± SD, and the sample size of each group was 9. ns *p* > 0.05 and ^∗∗∗^*p* < 0.001.

TUNEL staining was used to detect apoptotic cells in wound tissues. Apoptotic cells were marked pink in merged images. Results showed that the apoptosis indexes of the control and the LTS group were notably higher than the LTH group and the IIH group. Overall, hydrogen-rich saline treatment can effectively reduce the cell apoptosis of wound tissues (Figures 3(a) and 3(c)).

### 3.5. Hydrogen-Rich Saline Treatment Increased the Expression of Nrf-2

Immunohistochemical staining was used to detect the expression of Nrf-2; the dark brown stained tissue represents positive staining. The ratio of positive staining in the LTH group and the IIH group was notably higher than the control and the LTS group, which means the LTH group and the IIH group had more expression of Nrf-2 (Figures 3(b) and 3(d)).

### 3.6. Hydrogen-Rich Medium Reduced H_2_O_2_-Mediated Oxidative Stress

**Figure 4:**
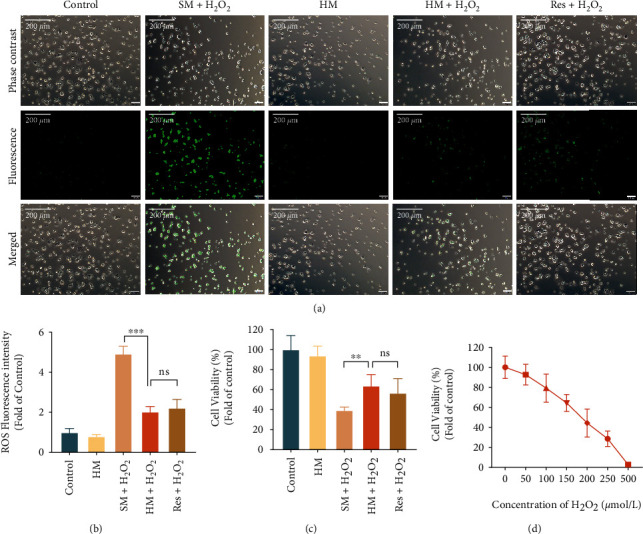
The oxidative stimulation of H_2_O_2_ and the antioxidation of hydrogen-rich medium. (a) Cells of each group were shown under light microscope, fluorescence microscope, and merged scope (Magnification: 100x). The green highlight represents ROS fluorescence. (b) The ROS fluorescence intensity in each group fold of the control. (c) The cell viability of each group with different treatment. (d) The cell viability with different H_2_O_2_ concentration treatment. (Fold of 0 *μ*mol/L). The results are expressed as mean ± SD and the sample size of each group was 6. ns *p* > 0.05, ^∗∗^*p* < 0.01, and ^∗∗∗^*p* < 0.001.

200 *μ*mol/L was determined as the concentration to provide oxidative stress stimulation (Figure 4(d)). We analyzed cellular ROS levels using DCFDA, which can fluoresce when oxidized by ROS. The results showed that H_2_O_2_ notably increased the level of ROS in cells, while this level was hugely reduced by hydrogen-rich medium pretreatment. There was no significant difference in cellular ROS level between the HM+H_2_O_2_ group and the Res+H_2_O_2_ group (Figures 4(a) and 4(b)). In CCK-8 experiment, we found that cell viability decreased significantly after H_2_O_2_ stimulation. The viability of cells pretreated with hydrogen-rich medium and resveratrol was higher than cells cultured in standard medium, that is, hydrogen-rich medium and resveratrol could resist oxidative stress in a certain degree (Figure 4(c)). In summary, hydrogen-rich medium pretreatment can alleviate H_2_O_2_-mediated oxidative stress.

### 3.7. Hydrogen Increased the Expression of Nrf-2 and HO-1

**Figure 5:**
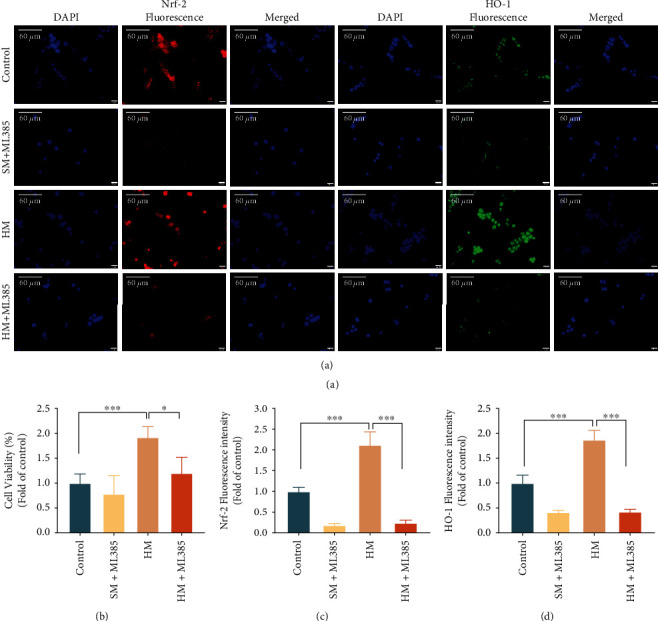
Hydrogen-rich medium functioned through Nrf-2/HO-1 signal pathway. (a) The immunofluorescence images of Nrf-2 and HO-1. (b) The cell viability of each group with different treatments. (c) The fluorescence intensity of Nrf-2 (d) The fluorescence intensity of HO-1. The results are expressed as mean ± SD, and the sample size of each group was 6. ^∗^*p* < 0.05 and ^∗∗∗^*p* < 0.001.

After H_2_O_2_ stimulation, the Nrf-2 fluorescence intensity of the HM group was strong and mostly expressed in the nucleus. The Nrf-2 fluorescence intensity of the control was weaker than the HM group and mostly expressed outside the nucleus. The fluorescence intensity of the SM+ML385 group and the HM+ML385 group using Nrf-2 inhibitor was weak. The fluorescence intensity of HO-1 showed a similar distribution as Nrf-2 in each group (Figures 5(a), 5(c), and 5(d)). Meanwhile, cell viability test showed that the cell viability of the HM group was higher than the control. The cell viability of the HM+ML385 group was lower than the HM group (Figure 5(b)). These results suggest that hydrogen-rich medium may alleviate oxidative stress mediated by H_2_O_2_ through upregulating the expression of Nrf-2/HO-1.

## 4. Discussion

In this study, we found that the local treatment of hydrogen-rich saline significantly increased the wound healing rate and reduced the oxidative stress, inflammation, and the proportion of apoptotic cells in the in vivo experiments. The study also showed that the application of hydrogen-rich medium alleviated the oxidative stress caused by hydrogen peroxide in HaCat cells through reducing the production of ROS. Moreover, Nrf-2/HO-1 may be the potential pathway through which hydrogen effects.

Homeostatic ROS plays a role in redox signal transduction in wound healing. A delicate balance between ROS levels and the body's antioxidant capacity is important for wound healing [4]. In this study, we found that the difference in wound area between groups reached a peak around the 7th day. Further detection of oxidation indicators in wound tissues on the 7th day showed that the experimental groups with hydrogen application had less lipid peroxidation products and more endogenous antioxidants than the control, which confirmed that the application of hydrogen affected the balance of oxidation and antioxidation. In the early stage of wound healing, severe ischemia and hypoxia and increased cell oxygen consumption will destroy ROS homeostasis, resulting in cell and tissue damage, and will finally delay the wound healing. In this stage, hydrogen may take advantage of its selective antioxidation to neutralize some strong oxidizing ROS and then restore the balance between ROS and antioxidants [41]. As a result, oxidative stress is alleviated, and the wound healing process is accelerated. In addition, mild inflammation is positive for wound healing via stress reaction activation and bacterial resistance [8]. However, excessive inflammation may aggravate oxidative stress [5]. In turn, oxidative stress can aggravate inflammation by activating macrophages and neutrophils to release inflammatory mediators such as tumor necrosis factors and interleukins [42]. This study indicates that the application of hydrogen can decrease the expression of proinflammatory cytokines (IL-1 *β*, IL-6, TNF- *α*, Caspase-1, and ICAM-1). We speculate that stress-induced inflammation is the major part where hydrogen effects its role. It is widely accepted that apoptosis is a type of programmed cell death, which will intensify ischemic injury and necrosis [43]. Increasing evidences suggest that ROS plays a vital role as a signal molecule in the whole apoptotic pathway [44]. In this study, the application of hydrogen reduced the cell apoptosis in wound tissues and inhibited the production of ROS in the cell experiment. It is reasonable to speculate that the application of hydrogen may reduce apoptosis by decreasing the production of ROS.

Nrf-2 is one of the critical regulators of antioxidant stress response elements. It regulates the protective and defensive responses in injuries and diseases, which plays a crucial role in antioxidant system and wound healing [45–47]. The main function of Nrf-2 in wound healing is to prevent excessive accumulation of endogenous ROS. A pronounced expression of Nrf-2 was found in macrophages and keratinocytes localized in hyperproliferative wound epithelium [48]. Under stress, the activated Nrf-2 in the cytoplasm transposes to the nucleus, where it binds to the promoter and upregulates the expression of HO-1. Additionally, HO-1, an anti-inflammatory and antioxidant enzyme, is also considered to be participated in wound healing [49]. This study showed that the application of hydrogen could increase the expression of HO-1. Current studies have confirmed that IL-10 is closely related to the anti-inflammatory effects of HO-1 [50, 51]. HO-1 reprograms macrophages from the M1 to the M2 phenotype, which produces IL-10 [52]. IL-10 is a negative-regulator cytokine that antagonizes the inflammatory response and regulates autoimmune activity [53]. We speculate that it is the reason of the upregulation of IL-10 in this study. To sum up, we believe that hydrogen can avoid excessive production of ROS by increasing the expression of Nrf-2 and HO-1, thus alleviating oxidative stress and promoting wound healing. IL-10 may be an important factor in the anti-inflammatory effect of hydrogen.

Hydrogen has been widely applicated by means of drinking, inhaling, and injection and all demonstrated effective [20, 36, 37]. Other than that, local application of hydrogen, an innovative approach of hydrogen application with multiple benefits, has attracted growing attention. It has been proved that hydrogen-rich eye drops have a protective effect on retinal ischemia-reperfusion injury by reducing retinal cell apoptosis, relieving oxidative stress, and activating glial cells [54]. Hydrogen bath can narrow the area of psoriasis lesions, alleviate itching symptoms, and dramatically improve the life quality of patients with psoriasis [21]. Interestingly, Tamaki et al. showed that by drinking hydrogen-rich water, the closure time of rat oral wound was shortened [22]. They also proved that the levels of proinflammatory cytokines were reduced and the expression of epidermal growth factor was improved in jaw tissues of rats by drinking hydrogen-rich water. It is reasonable to suspect that hydrogen-rich water may function by direct contact with the rat oral wound as well. Based on these discoveries, we assume that hydrogen might effect by taking advantage of its superior penetrability and diffusivity. In line with this hypothesis, we found in our study that the local treatment of hydrogen-rich saline remarkably promoted the wound healing process. Compared with intraperitoneal injection and direct inhalation, local treatment of hydrogen-rich saline is better in safety, effectiveness, and convenience, making it a potential therapy suitable for clinical application. However, how to control and maintain the concentration of hydrogen in the medium is a great challenge for hydrogen medicine at present. The concentration of hydrogen in the medium is low and difficult to control due to its superior penetrability. The usual method to raise concentration is to increase the pressure. But once the condition of high pressure is removed, the escape volume of hydrogen will increase. We have also encountered the problem during this research. Our solution is to make sure that all hydrogen products were used right after preparation. Even so, we can only restrict the concentration of hydrogen-rich saline used in each experiment in a narrow interval rather than a consistent value. The concentration of hydrogen-rich saline we detected was 0.7-0.8 mmol/L, which has been proved to be the effective concentration.

Specific limitation exists in our study. Pathological wound models like diabetic wound model and infected wound model were not included. Their mechanisms are complicated and influenced by many factors. We expect to explore the role of hydrogen on the standard physiological animal wound healing model to build a foundation for our follow-up work and provide potential mechanisms of pathological wound models. Moreover, to promote the healing process of physiological wounds is of great significance in clinical medicine. For example, after large area skin grafting, it is necessary to accelerate the wound healing of the skin donor sites so as to shorten the healing time, reduce the incidence of adverse events (e.g., wound infection and scar hyperplasia), and increase the utilization rate of skin donor sites.

## 5. Conclusion

This study demonstrated that local treatment of hydrogen-rich saline promoted wound healing by mitigating oxidative stress, alleviating apoptosis, and suppressing inflammation. Nrf-2/HO-1 signaling pathway might be the approach by which hydrogen-rich saline acted its antioxidant function. Considering the safety, effectiveness, and convenience of local treatment of hydrogen-rich saline, we pose strong faith that it may be a potential therapy to promote wound healing in clinical practice.

## Figures and Tables

**Table 1:** Primers sequence for quantitative real-time PCR.

Gene	Forward primer	Reverse primer
Caspase 1	5′-AATACAACCACTCGTACACGTC-3′	5′-AGCTCCAACCCTCGGAGAAA-3′
ICAM-1	5′-TCCGCTACCATCACCGTGTAT-3′	5′-TAGCCAGCACCGTGAATGTG-3′
IL-1*β*	5′-GCAACTGTTCCTGAACTCAACT-3′	5′-ATCTTTTGGGGTCCGTCAACT-3′
IL-6	5′-TAGTCCTTCCTACCCCAATTTCC-3′	5′-TTGGTCCTTAGCCACTCCTTC-3′
IL-10	5′-CTTACTGACTGGCATGAGGATCA-3′	5′-GCAGCTCTAGGAGCATGTGG-3′
TNF-*α*	5′-CAGGCGGTGCCTATGTCTC-3′	5′-CGATCACCCCGAAGTTCAGTAG-3′
GAPDH	5′-AGGTCGGTGTGAACGGATTTG-3′	5′-TGTAGACCATGTAGTTGAGGTCA-3′

## Data Availability

The article file contains all the datasets that support the conclusions of this study.
